# Hyperoxia in patients on venoarterial extracorporeal membrane oxygenation

**DOI:** 10.1186/s13054-022-04210-x

**Published:** 2022-10-27

**Authors:** Chenglong Li, Xiaomeng Wang, Xiaotong Hou

**Affiliations:** Center for Cardiac Intensive Care, Beijing Anzhen Hospital, Capital Medical University, 2 Anzhen Rd, Chaoyang District, Beijing, 100029 People’s Republic of China

To the editor,

We read with great interest the article by Moussa et al. which illustrated the association between early hyperoxia and 28-day mortality in patients on venoarterial extracorporeal membrane oxygenation (VA-ECMO) support for refractory cardiogenic shock [1]. Hyperoxia might increase oxidative stress, leading to worse outcomes [2]. Patients on VA-ECMO are the most affected by hyperoxia among the critically ill patients since they directly receive the oxygenated blood from VA-ECMO with PaO_2_ higher than 300 mmHg [3].

Moussa et al. analyzed the data of 430 patients from a bi-center cohort. They found that the mean daily peak, absolute peak, and overall mean PaO_2_ values 48 h after admission were significantly higher in non-survivors than survivors. After inverse probability weighting, the result remained the same. And, there was a dose effect of the PaO_2_ increase on the risk of 28-day mortality. One of the interpretations could be that exposure to hyperoxia results in an inappropriate inflammatory response. This could provide a theoretical basis for the management strategy of refractory cardiogenic shock. However, a hidden confounder might affect the interpretation of the results.

In the femo-femoral VA-ECMO, the retrograde blood flow produced by VA-ECMO and the anterograde blood flow produced by the patient's heart would meet in an aortic transition point, as the author mentioned. But the level of the transition point depended on the cardiac output (CO) [4]. When the cardiac contractility improves, and the CO is enough, the transition point may locate in the distal to the left subclavian artery. On the other hand, when the cardiac contractility is poor and the CO is low, the transition point may be proximal to the cephalic brachial artery. In other words, the cardiac function and CO decide the blood in the right radial artery where it comes from, the ECMO or the native heart.

Because PaO_2_ in the post-oxygenator is much higher than in the pulmonary vein, the blood gas analysis (BGA) taken from the right radial artery (where the study took the blood sample) was influenced to a large extent by the source of blood. If the blood comes from ECMO, there is a high PaO_2_ and vice versa. So, with this hypothesis, we could explain that the patient who presented with a higher PaO_2_ might suffer a worse cardiac function and be associated with mortality.

The indirect indicator of cardiac function is if the patient can successfully wean from VA-ECMO. We test this hypothesis with data in our center. From January 2017 to September 2022, 340 patients receiving femo-femoral VA-ECMO for cardiogenic shock whose ECMO duration was longer than 24 h were enrolled. Two hundred and sixteen (63.5%) patients were weaned from ECMO successfully. The in-hospital mortality was 52.4%. The 24-h (after ECMO start) BGA was taken from the right radial artery and analyzed. The patients were divided by if the 24-h PaO_2_ was higher than 200 mmHg. The outcomes are shown in the table (Table 1).

**Table 1:** Patients outcomes by PaO_2_ > 200 mmHg or PaO_2_ ≤ 200 mmHg

	All patients			Patients weaned from ECMO		
	PaO_2_ > 200 mmHg (*n* = 94)	PaO_2_ ≤ 200 mmHg (*n* = 246)	*P* value	PaO_2_ > 200 mmHg (*n* = 46)	PaO_2_ ≤ 200 mmHg (*n* = 170)	*P* value
In-hospital mortality	60 (63.8%)	118 (48.0%)	0.009	12 (26.1%)	42 (24.7%)	0.848
ECMO weaning*	46 (48.9%)	170 (69.1%)	0.001	–	–	–

*Successful ECMO weaning was defined by the lack of obvious hemodynamic deterioration for at least 48 h after the removal of the ECMO support

The categorical variables are compared with the chi-square test

Our results are similar to Moussa et al., finding that PaO_2_ > 200 mmHg was associated with mortality. There was a significant negative relationship between PaO_2_ and ECMO weaning (OR = 0.965 every ten mmHg, *p* = 0.004 by single variable logistic regression). The patients with higher PaO_2_ had a lower probability of weaning from ECMO (Table 1). In addition, we did not find any association between PaO_2_ and mortality among the patients who had already weaned from ECMO. This means cardiac function may be the major confounder in the evaluation of hyperoxia’s impact on patients' outcomes. The direct method is to test the relationship between CO and PaO_2_, but CO is difficult to acquire in the early stage of ECMO support.

Future translational research should focus on the role of oxidative stress involved in the development and progression of clinical and experimental heart failure [5]. Potential confounding that cannot easily be addressed in clinical studies may be answerable in bench experiments. More studies are needed, leading to novel therapeutic strategies in heart recovery.

## Data Availability

The datasets used in the present study are available from the first author and corresponding authors on reasonable request.
